# Next generation biobanking ontology: introducing–omics contextual data to biobanking ontology

**DOI:** 10.1093/bioadv/vbaf131

**Published:** 2025-08-07

**Authors:** Dalia Alghamdi, Damion M Dooley, Mannar Samman, Ali AlFaiz, William W L Hsiao

**Affiliations:** Bioinformatics Graduate Program, University of British Columbia, Genome Sciences Centre, BC Cancer, Vancouver, BC V5T 4S6, Canada; Centre for Infectious Disease Genomics and One Health (CIDGOH), Faculty of Health Sciences, Simon Fraser University, Burnaby, BC V5A 1S6, Canada; Executive Administration of Research Center, King Fahad Medical City, Riyadh 12231, Saudi Arabia; Centre for Infectious Disease Genomics and One Health (CIDGOH), Faculty of Health Sciences, Simon Fraser University, Burnaby, BC V5A 1S6, Canada; Clinical Pathology Department, King Fahad Medical City, Riyadh 12231, Saudi Arabia; Executive Administration of Research Center, King Fahad Medical City, Riyadh 12231, Saudi Arabia; Bioinformatics Graduate Program, University of British Columbia, Genome Sciences Centre, BC Cancer, Vancouver, BC V5T 4S6, Canada; Centre for Infectious Disease Genomics and One Health (CIDGOH), Faculty of Health Sciences, Simon Fraser University, Burnaby, BC V5A 1S6, Canada

## Abstract

**Motivation:**

With improvements in high throughput sequencing technologies and the constant generation of large biomedical datasets, biobanks increasingly take on the role of managing and delivering not just specimens but also specimen-derived data and associated contextual data. However, reusing data from different biobanks is challenged by incompatible data representations. Contextual data describing biobank resources often contains unstructured textual information incompatible with computational processes such as automated data discovery and integration. Therefore, a consistent and comprehensive contextual data framework is needed to increase discovery, reusability, and integrability across data sources.

**Results:**

The next generation biobanking ontology is an open-source application ontology representing omics contextual data, licensed under the Creative Commons 4.0 license. The ontology focuses on capturing information about three main activities: wet bench analysis used to generate omics data, bioinformatics analysis used to process and interpret data, and data management. In this paper, we demonstrated the use of the ontology to add semantic statements to real-life use cases and query data previously stored in unstructured textual format.

**Availability and implementation:**

NGBO is freely available at https://github.com/Dalalghamdi/NGBO, and accessible from OLS https://www.ebi.ac.uk/ols4/ontologies/ngbo.

## 1 Introduction

In the last couple of decades, using omic technologies like genomics and transcriptomics to understand human disease mechanisms has been highly successful (Karczewski and Snyder 2018). These studies have generated extensive biological materials (i.e. biospecimens) and data (i.e. contextual data such as clinical and demographic information, and specimen-derived data, such as sequence data) to enable broader research inquiries into basic, applied, and personalized health challenges (Libbrecht and Noble 2015). The generated data and specimens have become invaluable in fostering innovation, including their secondary use for new discoveries (Malsagova *et al.* 2020). As more studies expand the potential for these resources in advancing health-related research, researchers are increasingly interested in banking and reusing biospecimens, genetic materials, and derivative datasets.

Biobanks, once simple repositories for biospecimens, have evolved into complex organizations that collect not only biological materials but also a variety of datasets, including genetic profiles, omics datasets, and clinical records (Paskal *et al.* 2018). As biobanks evolve, they increasingly overlap with data repositories—once seen as distinct—which has exposed fragmented data structures and standards that complicate integration. This overlap has exposed fragmented data structures and standards that inadequately address the integration of these different data types. Effectively managing this data is crucial for enabling the discovery and integration of heterogeneous datasets, and as biobanks grow in complexity, agile data management systems and standards are essential to support their role in advancing biomedical research (Griffiths *et al.* 2017).

Various data standardization initiatives, including minimum information checklists and best practices, have been developed to address these integration challenges. One notable initiative is the biobanking and biomolecular resources research infrastructure (BBMRI) consortium, which established MIABIS (minimum information about BIobank data sharing) to ensure consistent categorization across biobanks, facilitating data integration and comparison (Norlin *et al.* 2012, Eklund *et al.* 2020, 2024). Another effort is ISBER’s Best Practices (Wilde 2023), which provides comprehensive guidelines for managing biorepositories, including documentation and data management. These standards cover essential areas such as informed consent, sample collection protocols, and quality assurance, as detailed in Supplementary Table S1.

Despite these efforts, the adoption of standards like MIABIS and ISBER remains limited, largely due to challenges such as significant infrastructure requirements, integration complexities, and the need for domain-specific extensions (Brancato *et al.* 2024). Even when implemented, inconsistencies in contextual data documentation often undermine their effectiveness, making it difficult to achieve comprehensive and interoperable datasets. Addressing these gaps with more computationally indexed and interoperable standards is crucial for improving the reliability and utility of biobank data. For example, incomplete metadata in the UK Biobank, including inconsistencies in lifestyle factors like alcohol consumption and physical activity, highlights the ongoing difficulties in maintaining high-quality, consistent data (Coppola *et al.* 2019, Bassett *et al.* 2024). In this paper, we primarily use the term “contextual data” to describe descriptive information associated with specimens. However, some referenced sources, including standards and repositories like the UK Biobank, refer to similar concepts as “metadata.” For consistency with these sources, both terms appear interchangeably in this document.

**Figure 1.**
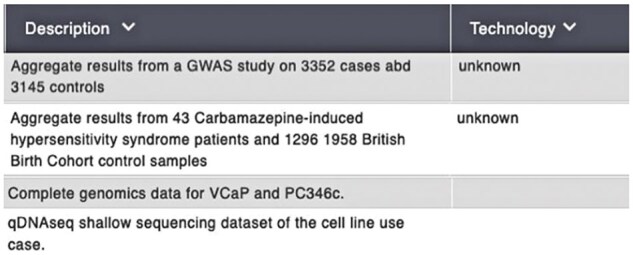
Missing contextual data fields across genomics records in EGA datasets. Screenshot taken by the authors from the European Genome-phenome Archive website (https://ega-archive.org).

These metadata challenges are even more pronounced in omics research, where current standards often fall short of the specific requirements for managing omics data comprehensively. Public databases such as the European Genome-phenome Archive (EGA) face similar issues, particularly in areas like sequencing technologies and sample preparation methods. Figure 1 highlights missing contextual data fields in EGA datasets, such as the lack of sequencing technology information. This absence creates challenges for data users, making it difficult to query data by sequencing platforms and to analyze data from different platforms. Properly annotating the technological details is essential for effective data discovery, retrieval, and analysis.

To address the issue of missing contextual data, we developed a suggested information checklist based on the College of American Pathologists (CAP) documentation requirements, as presented by Aziz *et al.* (2015). CAP is recognized as a leading organization in laboratory quality assurance and pathology and known for its rigorous standards and guidelines that ensure the accuracy, reliability, and integrity of laboratory data. By adopting CAP documentation requirements, we aim to ensure that the biobanking data is comprehensive and precise, reducing the risk of errors while enhancing data reusability. In the ideal case, the contextual data should provide all necessary information to re-analyze available data, on the one hand, and should enable researchers to reuse data in a broader context going beyond individual studies, on the other hand. However, semantic ambiguity—where terms like “allele frequency” may mean different things—can lead to misinterpretation, the term could either refer to the frequency of a specific allele within a studied cohort or to its global population frequency from a reference database like 1000 Genomes.

Using ontologies, the formal logical representations of terms, prevents unnecessary complications that can lead to mistakes during data management and retrieval processes (Smaili *et al.* 2019). Ontologies resolve ambiguity by formally defining terms, ensuring consistent interpretation across systems. This eliminates multiple interpretations of the same term, allowing data to be uniformly understood across systems and studies. Ontologies support the implementation of the FAIR data principles (Jacobsen *et al.* 2020) FAIR principles—Findable, Accessible, Interoperable, and Reusable—provide a critical framework to address these needs, ensuring that data is properly organized, easy to locate, and readily available for use across different studies and platforms.

Several ontologies have been developed to support biobanking, and prior work has laid the foundation for semantic integration in this field. The initial use of ontologies in biobanking began in 2014 with the introduction of the Ontologized MIABIS (OMIABIS) which is used in BBMRI infrastructure (Brochhausen *et al.* 2013). OMIABIS was later extended and combined with biobanking ontology, forming a novel Ontology for Biobanking (OBIB) (Brochhausen *et al.* 2016). Ontologies, unlike traditional databases that focus on structured storage and retrieval, enable the semantic integration of complex, interdisciplinary datasets. Rather than merely storing data, they represent entities described within the data and define relationships between them. These relationships, particularly in the web ontology language (OWL), hold between values rather than raw data elements, allowing for logical inferencing and interoperability across heterogeneous sources. Often used alongside triple stores, ontologies enhance data discovery and interoperability across heterogeneous sources. For example, researchers can effectively connect genetic variants in biobank datasets with functional annotations available in the gene ontology (GO) (Ashburner *et al.* 2000), as implemented in the Ensembl database (Hubbard *et al.* 2002).

In this paper, we present the next generation biobanking ontology (NGBO), an open biobanking ontology developed based on the open biological and biomedical ontology (OBO) Foundry Principles. NGBO has been evaluated and accepted by the OBO Foundry and is accessible through the ontology lookup service (OLS) (Côté *et al.* 2006). NGBO uses the Saudi human genome project (SHGP) as a proof-of-concept use case, demonstrating how semantic web technologies can improve querying and integrating biobank specimens and associated data. Another proof-of-concept use case involves grant-funded projects in Riyadh’s Second Health Cluster (R2), a network of healthcare hospitals and centers that also focuses on biomedical research. These projects generate diverse datasets with some biospecimens deposited in the R2-associated biobank, dedicated to preserving and managing specimens for long-term research use. This biobank supports clinical and research activities by ensuring sample quality and accessibility, making it an ideal setting for demonstrating the capabilities of NGBO in managing and integrating biobank data.

## 2 Methods

**Figure 2.**
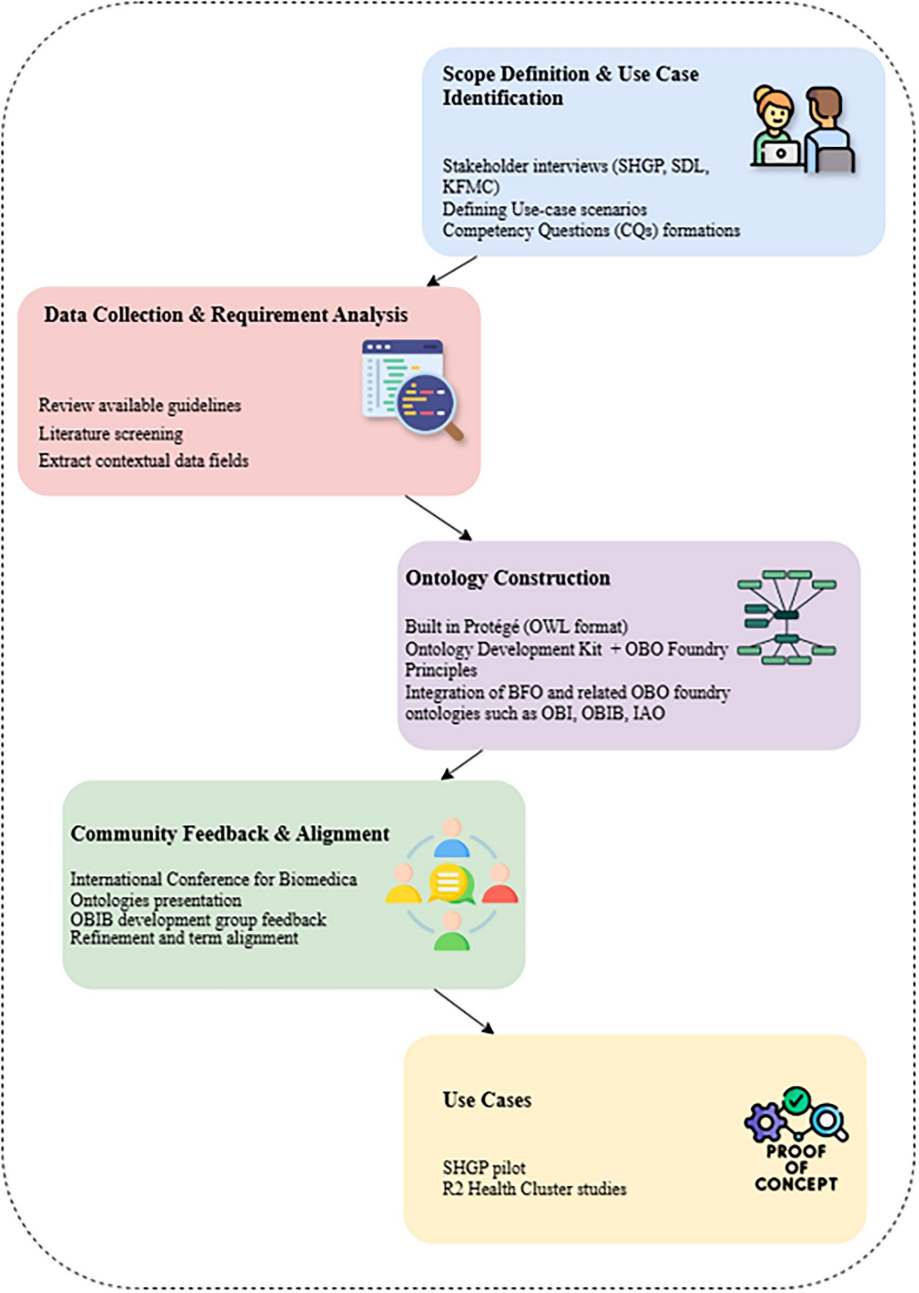
Overview of the NGBO development process. This figure summarizes the major steps taken in developing NGBO, including scope definition, data requirement analysis, specification development, ontology construction, and community alignment. It provides a high-level view of the methodology presented in detail in this section.

An overview of the NGBO development process—from scope definition to technical implementation and community feedback—is presented in Fig. 2. This figure provides a high-level summary of the methodology described throughout this section.

### 2.1 Defining NGBO scope

We approached the development of NGBO by first answering foundational questions about ontology’s purpose, scope, intended end-users, and usage (see Table 1) following the approach outlined by Suárez-Figueroa *et al.* (2009). To define the scope and delimit the knowledge (i.e. specific data fields and relations) represented by NGBO, we initiated discussions with domain experts at Saudi Diagnostic Labs (SDL) and King Fahad Medical City (KFMC), both partners of the SHGP. These discussions involved researchers experienced in requesting specimen-derived data from repositories and helped us identify general scenarios. Based on these discussions, we clarified the need for NGBO and proposed several use-case scenarios. Following this, we formulated a set of competency questions (CQs) expressed in natural language to evaluate the scope and usage of the ontology, which was formalized into description logic (DL) queries according to the language used to represent the ontology. The CQs were used to verify the ontology’s requirements by ensuring that it could retrieve correct results, thereby validating its effectiveness and completeness in meeting the defined needs. The full list of CQs can be found in Supplementary CQ.

**Table 1.** NGBO specification.

NGBO specification	
Purpose	NGBO is developed to integrate contextual data, initially incorporating genomics and transcriptomics with existing biobanking ontologies. It aims to provide semantic services for biobank omics data management, discovery, and reuse.
Scope	This ontology includes descriptions of real-world physical objects such as specimens, computerized objects such as datasets, and planned processes and related specifications. Although biospecimens are collected from various sources (e.g. humans, animals, plants, and the environment), the current NGBO scope focuses on human biobanks. The ontology can be extended to other species depending on use-case requirements.
Intended end user	**User 1:** Bioinformaticians and computational biologists who require integration and analysis of -omics data from various biobank specimens. **User 2:** Data curators and managers are responsible for maintaining biobank databases and ensuring data quality and interoperability. **User 3:** Policy makers and regulatory bodies who need standardized and accessible contextual data about omics dataset to inform health policies and regulations. **User 4:** Research institutions and consortia collaborating to federate biobank data across multiple repositories for large-scale studies.
Intended use-case	**Use case 1:** Facilitate the discovery and retrieval of specific biobank specimens and associated omics data through standardized contextual data integration. **Use case 2:** Improve the quality and consistency of biobank data by providing standardized templates and guidelines for data curation. **Use case 3:** Ensure that biobank data meets regulatory standards and is uniformly reported. **Use case 4:** Standardizing data representation, which is crucial for effective data federation. This will enable large-scale studies by combining data from various sources, increasing the statistical power and scope of research findings.

### 2.2 Ontology development process

Next, based on defined scope and usage, we cataloged and studied relevant specifications and existing resources. This included reviewing a selection of public repository requirements, such as those from the National Center for biotechnology information (NCBI) BioSample, as well as requirements from the CAP laboratory guidelines, a particular attention was given to the CAP molecular pathology documentation requirements (Aziz *et al.* 2015) to ensure NGBO supports essential quality assurance and traceability practices. This involved modeling processes related to both wet bench assays and bioinformatics pipelines. We also examined other available guidelines, such as the minimum information about microarray experiments (MIAME), the minimum information about any (x) sequences. The results of this analysis are presented in Supplementary Table S2. Additionally, we considered relevant aspects of the American College for Medical Genetics and Genomics (ACMG) guidelines, such as variant classification guidelines, and clinical laboratory reports from KFMC. However, this initial review did not provide sufficient data fields to fully address the NGBO use-case scenarios because the reviewed guidelines and reports were either too general or did not cover specific aspects of biobank data and omics integration and contextual data requirements unique to the NGBO framework. As a result, we needed to expand our data sources for a more comprehensive analysis. To address this gap, we screened public contextual data from a selection of journal publications from 2017 to 2019 by searching PubMed using a snowball sampling method. We began with relevant papers and selected additional ones based on their titles, abstracts, and references, continuing until we gathered enough data to address the NGBO specification questions. Keywords used in our search included “biobank data management,” “omics data integration,” “biobank guidelines,” “data standardization,” “minimum information requirements,” and “specimen metadata.” The results of this keyword search can be found in Supplementary Table S3.

From this expanded analysis, we identified a comprehensive list of contextual data required for accurately reporting the experimental procedures involved in wet bench and bioinformatics analysis. This list formed the foundation for constructing the minimum information checklist, which includes essential data fields for consistent and transparent reporting. The distinction here is that the comprehensive list encompasses all relevant contextual data, while the minimum information checklist specifically targets the key elements needed to ensure interoperability and standardization.

### 2.3 Technical development

To manage the development of NGBO, we set up a GitHub repository using the Ontology Development Kit (ODK). ODK is a toolkit designed for creating, maintaining, and standardizing biomedical ontologies, offering standardized, customizable, and automatically executable workflows for ontology lifecycle management (Matentzoglu *et al.* 2023, Matentzoglu and INCATools n.d.). We used the Protégé ontology editor to build the ontology in the OWL format (World Wide Web Consortium n.d.). To integrate external ontologies and guide term relationships, we adhered to OBO Foundry Principles (Smith *et al.* 2007). Basic formal ontology (BFO) (Arp *et al.* 2015) was utilized as the top-level ontology for NGBO development.

To avoid redundant representations, we carefully selected and imported preexisting terms from well-maintained ontologies. These ontologies are annotated with proper Permanent URLs (PURL)s, labels, and textual and logical definitions. For example, we imported terms from the Ontology for Biomedical Investigations (OBI) to describe entities involved in biomedical research, including material entities, processes, and protocols, using OWL import (Bandrowski *et al.* 2016). Additionally, from OBIB (Brochhausen *et al.* 2016), we imported terms to describe specimens, donors, and specimen management (Brochhausen *et al.* 2016). Information Artifact Ontology (IAO) (Zheng n.d.) is another relevant ontology used as a mid-level ontology and includes terms describing identifiers, software solutions, and documents. In total, terms from twenty ontologies were imported. The top five ontologies by term count are CL (660 terms), OBI (659 terms), OBIB (167 terms), IAO (70 terms), and RO (68 terms), collectively accounting for 83.3% of all imported terms. A full breakdown is presented in Supplementary Fig. S1.

As part of NGBO’s technical design, we implemented features for specimen tracking and traceability by modeling key process relationships (e.g. has performer, has specified input/output, part of) and introducing object properties such as trace from and track to. These relations support bidirectional tracing of specimen and data flow across experimental and computational processes, fulfilling traceability requirements from standards like CAP. We also introduced basic modeling of data privacy and access control, using semantic roles (e.g. authorized user, data owner) and object properties like accessed by to demonstrate how NGBO could support controlled data access within federated biobank environments.

Input from the broader OBO Foundry community was solicited during the international conference on biomedical ontologies (ICBO) 2019, where NGBO was first presented. The feedback from this conference played a critical role in refining NGBO’s scope and alignment with existing ontologies. Additionally, NGBO benefited from sustained discussions with the OBIB development group over multiple months, particularly regarding the modeling of specific terms and the integration of OBIB concepts within NGBO. These discussions facilitated the refinement of NGBO’s structure and ensured that NGBO-defined terms align with existing ontologies within the OBO framework. As part of this alignment, there was a consensus that NGBO, as an application ontology, would contribute newly defined terms to relevant domain ontologies, reinforcing its interoperability within broader biobanking and biomedical ontology ecosystems. The OBIB group was generally supportive of NGBO’s approach, particularly in its efforts to enhance semantic integration across biobank data resources.

## 3 Results

NGBO is an open-source application ontology written in OWL and licensed under the Creative Commons Attribution 4.0 International Public License. It is deposited in a public GitHub repository, where ontology releases and term requests are maintained, and accessed by OLS at https://www.ebi.ac.uk/ols4/ontologies/ngbo. The latest release, published on September 10th, 2024, contains 1,515 classes, 252 properties, 165 NGBO-defined classes, and 1,942 logical axioms and relations.

The review of existing standards revealed significant gaps in satisfying NGBO use cases. Supplementary Table S2 provides a comprehensive overview of how various standards align with NGBO, highlighting specific gaps and overlaps. Biobanking and specimen handling standards often lack the granularity needed to capture details on sequencing platform versions, quality control metrics, and bioinformatics pipelines, critical for omics data integration. Conversely, while sequencing standards like MIxS capture these details, they do not address specimen handling or consent information, creating challenges for automated data integration.

### 3.1 Ontologizing contextual data structure for wet bench analysis

**Figure 3.**
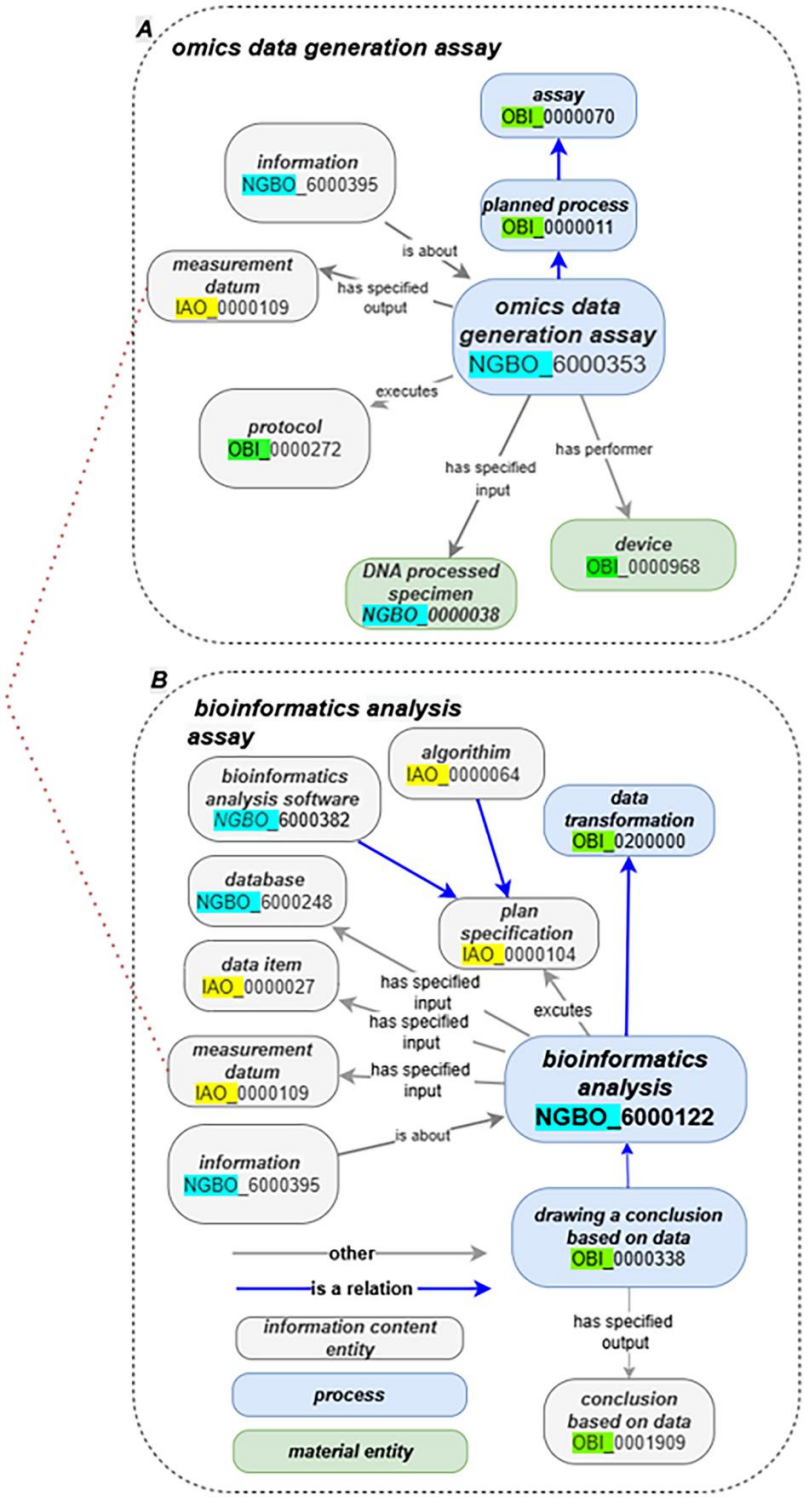
(A) Illustration of the omics data generation assay. (B) Illustration of the bioinformatics analysis and a selection of possible pipeline components. This figure also demonstrates how omics data generation is directly connected to the bioinformatics analysis workflow. Specifically, the measurement datum produced as the output of the data generation assay serves as the input for subsequent computational analysis. This highlights the continuity and integration between laboratory-based data production and downstream bioinformatics processing.

Given the rapid evolution of sequencing technologies, ensuring traceability for each processing step is crucial (Micheel *et al.* 2012). NGBO defines an omics data generation assay as “a planned process that generates omics data through a wet bench process, such as sequencing techniques.” Each NGBO-defined entity related to omics data generation assays include multiple participants and concepts, as outlined in the suggested information checklist for omics data generation assays (see Supplementary Table S4). Each assay specifies its inputs and outputs to achieve its objectives. For instance, a “DNA processed specimen”—defined as “a processed specimen which is the output of preparing a DNA sample for further analysis”—is captured as an input of the process using the “has specified input” relation. Similarly, the measurement datum “output data file”—defined as “A data file which contains the output of data transformation process”—is captured as an output using the “has specified output” relation. Figure 3A shows how NGBO integrates and manages information describing the wet bench process.

### 3.2 Ontologizing contextual data structure for dry bench analysis

Other NGBO development efforts focus on concept curation for bioinformatics analysis and software. NGBO captures details such as process participants, specified inputs and outputs, and execution information (including executor name, date, and run identifier [ID]) for bioinformatics processes. It also incorporates plan specifications, including algorithms, protocols, and bioinformatics software. For instance, “bioinformatics analysis software” refers to software designed to extract meaningful information from molecular biology data. We propose a suggested minimum information checklist for bioinformatics software and single nucleotide polymorphism (SNP) reporting, detailed in Supplementary Tables S5 and S6).

NGBO models bioinformatics software components, including algorithms, databases, and protocols, and links these to wet lab processes. This connection enables bioinformatics analysis of specimen-derived data while maintaining traceability to the original specimen. Version traceability of bioinformatics analysis software is recorded using a “version number” datum and the “is about” relation

For example, NGBO captures command-line parameters, such as the “-A INT” flag used in the Burrows–Wheeler Aligner algorithm, which is crucial for data re-analysis. Additionally, NGBO augments data management activities, including de-identification, storage, and transfer, with detailed information on storage data and protocols. This comprehensive approach ensures robust management and integration of bioinformatics data as illustrated in Fig. 3B, enhancing reproducibility and data handling efficiency.

### 3.3 Implementation of CAP requirements in NGBO

NGBO was developed to comply with CAP standards for omics data generation assays and biobanking data management. It incorporates detailed specifications to ensure that biobanks can meet CAP’s molecular pathology documentation requirements as published in (Aziz *et al.* 2015) for quality assurance, validation, and traceability. Here are key examples of how NGBO supports CAP compliance:

1. Wet Bench Process Documentation: NGBO models critical elements such as sample preparation, library preparation, and sequencing, linking them to specific reagents, instruments, and software used. This ensures each step is documented, meeting CAP’s requirement for detailed Standard Operating Procedures (SOP)s and facilitating the traceability of processes.
2. NGBO provides a framework for documenting activities, capturing the entire workflow from specimen handling to data processing. This traceability is achieved through properties like “has specified input” and “has specified output,” allowing labs to maintain comprehensive records of all processes, as required by CAP. Later in the paper, the specimen tracking and traceability section provides a more detailed explanation of how NGBO supports comprehensive specimen traceability, using process properties like “has specified input” and “has specified output” to document every step and interaction in the specimen's lifecycle.
3. Bioinformatics Pipeline Documentation and Validation: NGBO models terms for tracking and documenting each step of the bioinformatics pipeline, including sequence alignment and variant calling. This supports CAP’s requirements for bioinformatics validation and version control, ensuring that all software components and configurations are traceable.

### 3.4 NGBO evaluation

#### 3.4.1 NGBO evaluation by OBO foundry

NGBO has been evaluated and accepted by the OBO Foundry after undergoing both automated checks and manual review by the OBO Foundry Operational Committee. Detailed discussion can be found in the OBO Foundry GitHub issue: https://github.com/OBOFoundry/OBOFoundry.github.io/issues/1819#issuecomment-1396905637. The evaluation included the ontology's title, ID space, repository location, contact information, issue tracker, license (CC-BY), and related OBO Foundry ontologies. NGBO adheres to key principles such as open access, unique URI, common format, and proper versioning. After its acceptance, NGBO was registered in the official OBO Foundry metadata registry, available at: https://github.com/OBOFoundry/OBOFoundry.github.io/blob/master/ontology/ngbo.md.

#### 3.4.2 NGBO evaluation by CQs and reasoners

To validate and test the adequacy of the ontology, we referred to the method used in a previous study (Brochhausen *et al.* 2013). DL queries over an OWL file populated with mock data instances similar to those in SHGP, and false positive and negative entries at the instance level. The mock data were imported into the NGBO OWL file using the Celfie plugin (Protégé Project Contributors n.d.). The DL queries are implementations of the previously proposed CQs.

When evaluating the quality of NGBO, we considered two principles:

1. Clarity: NGBO does not create any misinterpretation during query execution or reasoning.
2. Consistency: all individuals were accurately categorized when the reasoning procedure was completed.

Reasoning is deriving implicit facts from a set of given explicit facts. In NGBO, these facts are expressed in OWL and stored in resource description framework (RDF) triplestores. For example, the following fact: “A bioinformatics analysis executes an algorithm” can be expressed in an ontology at the class level as “bioinformatics analysis [NGBO:6000122] executes some algorithm [IAO:0000064],” while an instance of this fact involving a particular job run and software reference: “bioinformatics analysis X123 executes algorithm Y456” can be stored in a triplestore. A reasoner is a software application that uses an ontology to infer relationships that should hold between existing classes and their instances. A reasoner categorizes entities under ontological hierarchies and identifies contradictions. To validate whether or not NGBO is consistent, we used HermiT (Shearer *et al.* n.d.) and Pellet (Sirin *et al.* 2007) reasoners. Both reasoners confirmed the ontology’s consistency, with no logical contradictions detected in the ontology’s structure or axioms. To evaluate NGBO’s ability to answer the CQs, we ran DL queries (listed in Supplementary CQ) after reasoning. The results confirmed that NGBO successfully retrieved the expected outputs for all CQs, demonstrating that the ontology effectively meets its defined requirements for querying biobank data.

### 3.5 Use cases

#### 3.5.1 Enhancing data integration and discoverability in SHGP using NGBO

The SHGP, launched in 2014, aimed to create a comprehensive genetic database for the Saudi population. However, like many large-scale genomic initiatives, SHGP faced significant challenges in managing and integrating its diverse data types. Data generated from various bioinformatics tools, laboratory SOPs, and sequencing pipelines were stored in siloed systems, making discovery and reuse difficult.

**Figure 4.**
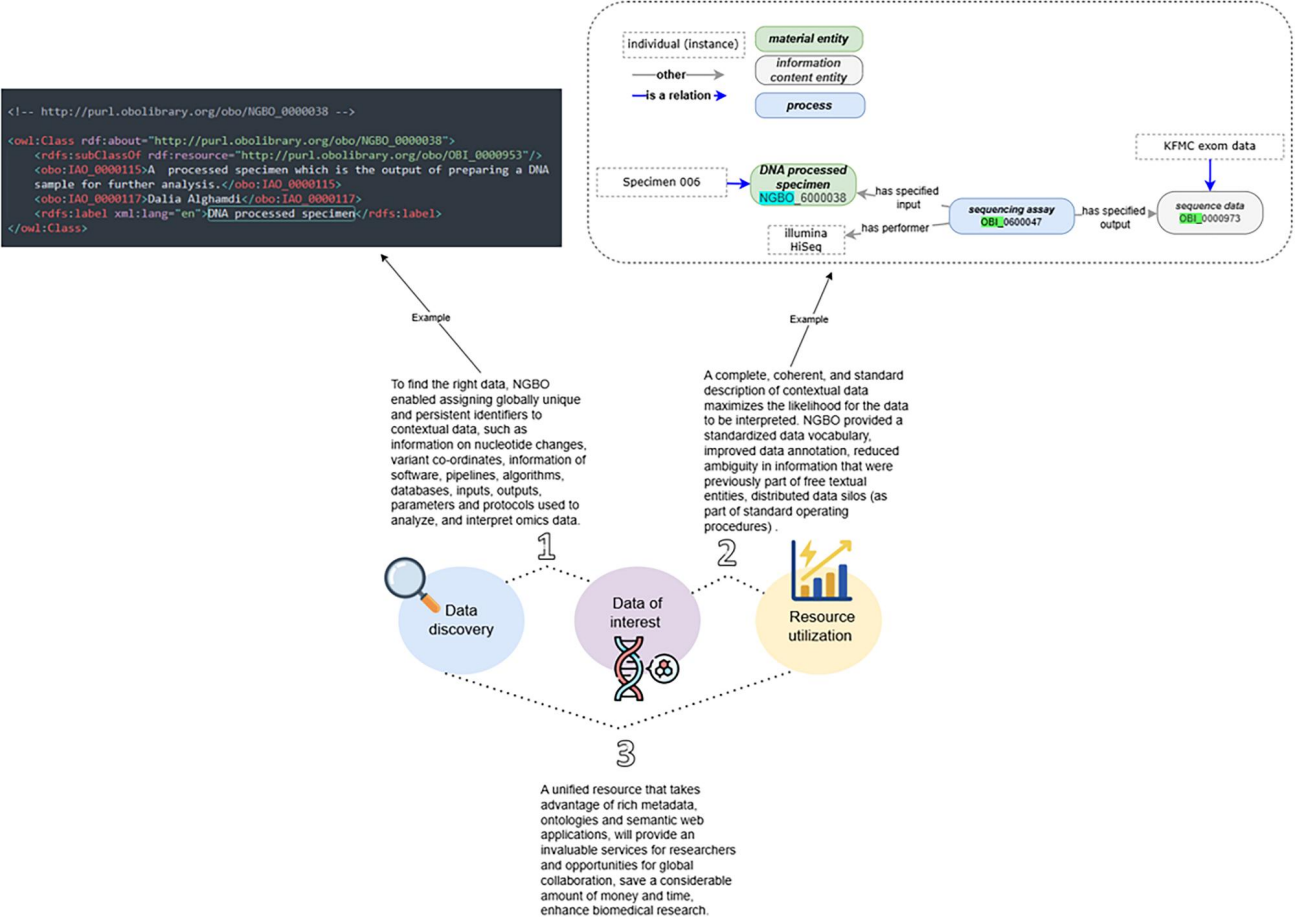
Illustration of the approach implemented using NGBO to improve the discovery of SHGP data and the FAIRness of the data.

NGBO addresses the challenge of integrating diverse data types by introducing a structured, ontology-based framework. For example, each entity is assigned a globally unique identifier, such as “DNA processed specimen” (NGBO_6000038). This specimen is linked to a “sequencing assay” (OBI_0600047), which captures critical contextual data about the processing, including the specified input (e.g. Specimen 006), the output “sequence data” (OBI_0000973), and the performer (e.g. Illumina HiSeq platform). These relationships, defined using ontology-based fields like “has specified input” and “has specified output,” ensure that each component in the biobank dataset is contextually connected. Figure 4 illustrates how NGBO enables comprehensive linkage among data entities, thereby supporting efficient data governance and fostering the reuse of genetic resources.

To evaluate NGBO’s impact, we manually assessed metadata terms used across SHGP sequencing submissions. Before annotation, 100% of entries documented library preparation and quality control information as unstructured free-text, making it difficult to retrieve or compare records across datasets. After applying NGBO, platform names were harmonized under a single controlled term, and both library preparation and quality control metadata were restructured using standardized ontology fields. This standardization enabled precise querying using DL queries introduced earlier in the manuscript by specimen type, platform, and processing protocol.

By employing this level of granularity and precision, NGBO enables researchers to perform targeted queries across datasets, locating data such as nucleotide changes and variant annotations tied to specific assays or specimen types. This structured approach not only aligns with the FAIR data principles, but also enhances resource utilization by facilitating seamless data discovery and integration across biorepository platforms. To further clarify these results, NGBO’s structured framework has transformed SHGP’s data discovery workflow, replacing manual searches with ontology-powered querying and enhancing metadata consistency across biobank records. Previously, there were numerous inconsistencies in specimen metadata, such as variations in terminologies used for sequencing platforms (e.g. “HiSeq 2500” vs. “Illumina HiSeq”), and missing quality control details in sequencing metadata. By enforcing standardized terminology and structured metadata representation, NGBO has significantly improved data integration, ensuring that sequencing data can be efficiently retrieved based on specimen and assay relationships. NGBO’s contributions to semantic interoperability and query automation are explicitly highlighted throughout the manuscript.

#### 3.5.2 Application of NGBO for managing datasets generated in grant-funded projects in R2

The R2 Health Cluster, which consists of 64 hospitals and research centers, generates diverse clinical and molecular datasets, including studies that generate omics data, with some study samples deposited into the cluster’s biobank. However, each institution often uses varying formats and terminologies to describe similar data types, such as genetic mutations, sample handling, and sequencing protocols, complicating data merging and analysis. NGBO addresses these issues by standardizing study designs, interventions, and data types, ensuring consistent descriptions across projects. This standardization supports better data integration, discovery, and reuse, while also improving traceability of data and samples, ensuring transparency and reproducibility across the cluster.

For instance, when expression Quantitative Trait Loci (eQTL) data is submitted to the R2 data governance team, NGBO assigns predefined identifiers to crucial data fields such as chromosome number (NGBO_6000476), Detected Gene Mutation (NGBO_6000439), and Expression Quantitative Trait Loci Data (NGBO_6000477). These identifiers ensure uniform representation for essential eQTL data fields while allowing partnering institutions to use different labels. NGBO also standardizes contextual data files for gene and SNP locations, which include information such as chromosome number, starting and ending loci, and SNP positions. Consistent SNP information is essential for accurate eQTL data analysis, as it enables the linkage between genetic variants and gene expression changes.

To date, NGBO has been used to standardize metadata for 38 grant-funded datasets submitted to the R2 Health Cluster. In addition to harmonizing metadata across bioinformatics and library preparation workflows, NGBO specifically addressed eight major SNP-related terms that required harmonization; these terms are listed in Supplementary Table S6. Prior to NGBO implementation, each dataset contained 5–9 inconsistencies in core fields such as SNP identifiers, variant representation formats, and gene symbols. After standardization using NGBO-defined terms and structure, all submissions aligned with cluster-wide metadata guidelines, and duplicate variant records due to inconsistent labels were fully resolved. This has significantly improved the efficiency of cross-study comparisons and enabled automated querying across research-generated datasets, with internal data governance committee feedback indicating a noticeable reduction in manual curation efforts.

### 3.6 Additional features

#### 3.6.1 Specimen tracking and traceability

The detailed documentation of all specimen processing activities is critical for many reasons, including accreditation and regulatory body requirements, collaboration and data reproducibility, and audibility. For example, CAP requires traceability of all standard operating protocols for DNA/RNA specimen preparation, library preparation, sequence generation, and bioinformatics analysis. NGBO includes terms describing specimen processing, material entities, and information entities, including information about designated team members or personnel who perform any specimen- or data-related activities.

To support end-to-end traceability, NGBO defines a set of object properties (relations) that describe how data and specimens flow through a series of processes. Central to this structure are the relations “trace from” and its inverse, “track to,” which enable bidirectional tracing of specimens and associated data. The relation “trace from” includes several sub-properties that represent specific aspects of workflow provenance. For example, “has performer” denotes a relation between a planned process and a continuant—such as a person, organization, or device—that performs the process. “has specified input” links a process to the material entity that serves as its input, while “has specified output” connects it to the entity it produces. Both are inverses of standard OWL properties and help capture input-output dependencies within processes. The relation “part of” is used to represent compositional relationships, such as a subprocess being part of a larger experimental protocol.

**Figure 5.**
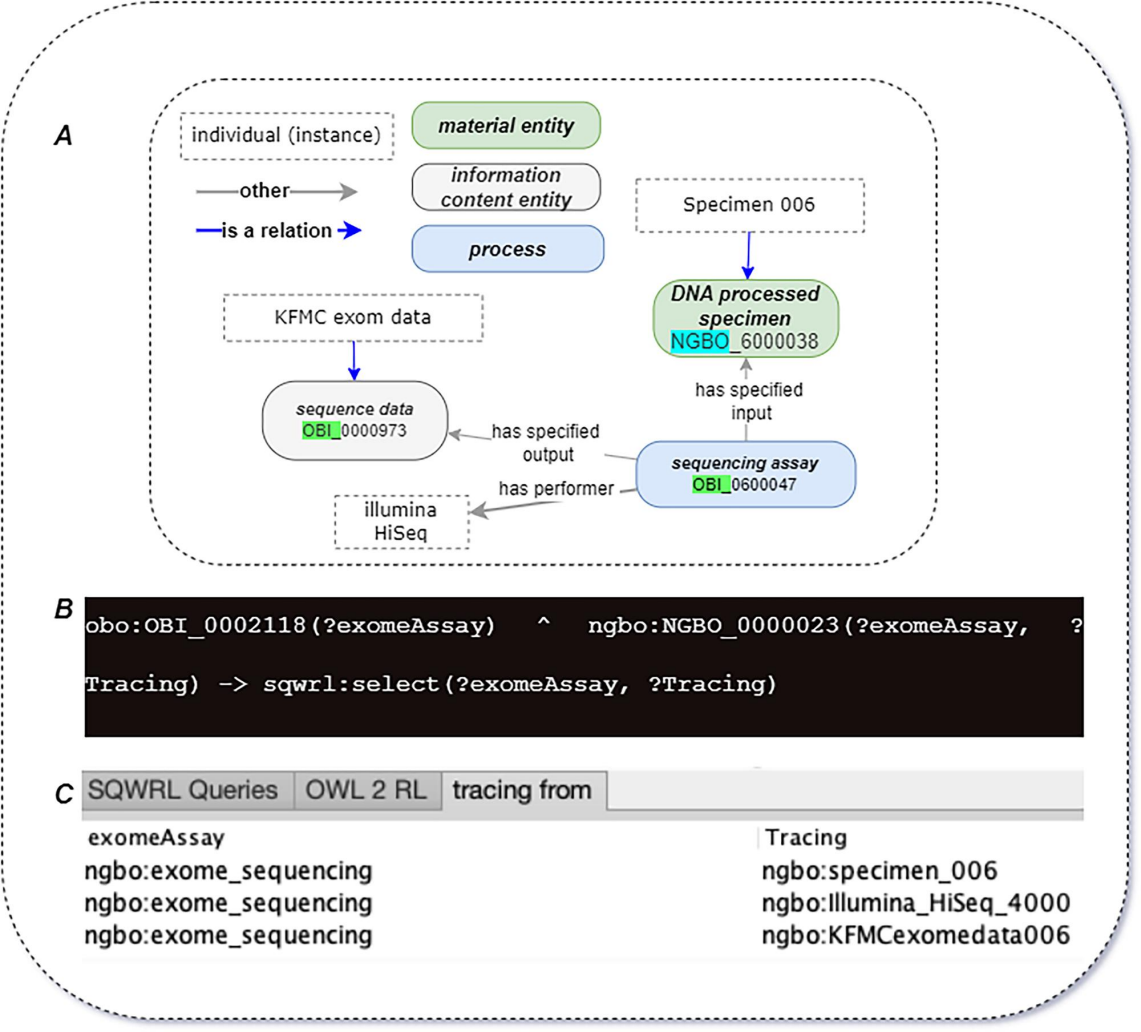
Using NGBO for specimen tracking and traceability. (A) An example of relations between individuals. (B) A top-down tracing SQWRL query using the NGBO_0000023, which denotes a “trace from” relationship. (C) Table of query results. The traceability results of the query are derived through an ontology reasoning system and selected in variable tracing. The variable “ExomeAssay” contains instances linked through the “trace from” relationship.

Conversely, the relation “track to” and its sub-properties serve as inverse properties of “trace from” and enable top-down exploration of how a dataset or output originated. When instances of processes, their inputs, outputs, and performers are explicitly represented, NGBO allows for ontology-based reasoning to infer and navigate complete chains of evidence. This facilitates bottom-up or top-down traceability using languages such as Semantic Query-Enhanced Web Rule Language (SQWRL). An example of this reasoning-based tracing approach is shown in Fig. 5.

#### 3.6.2 Data privacy and user access control

**Figure 6.**
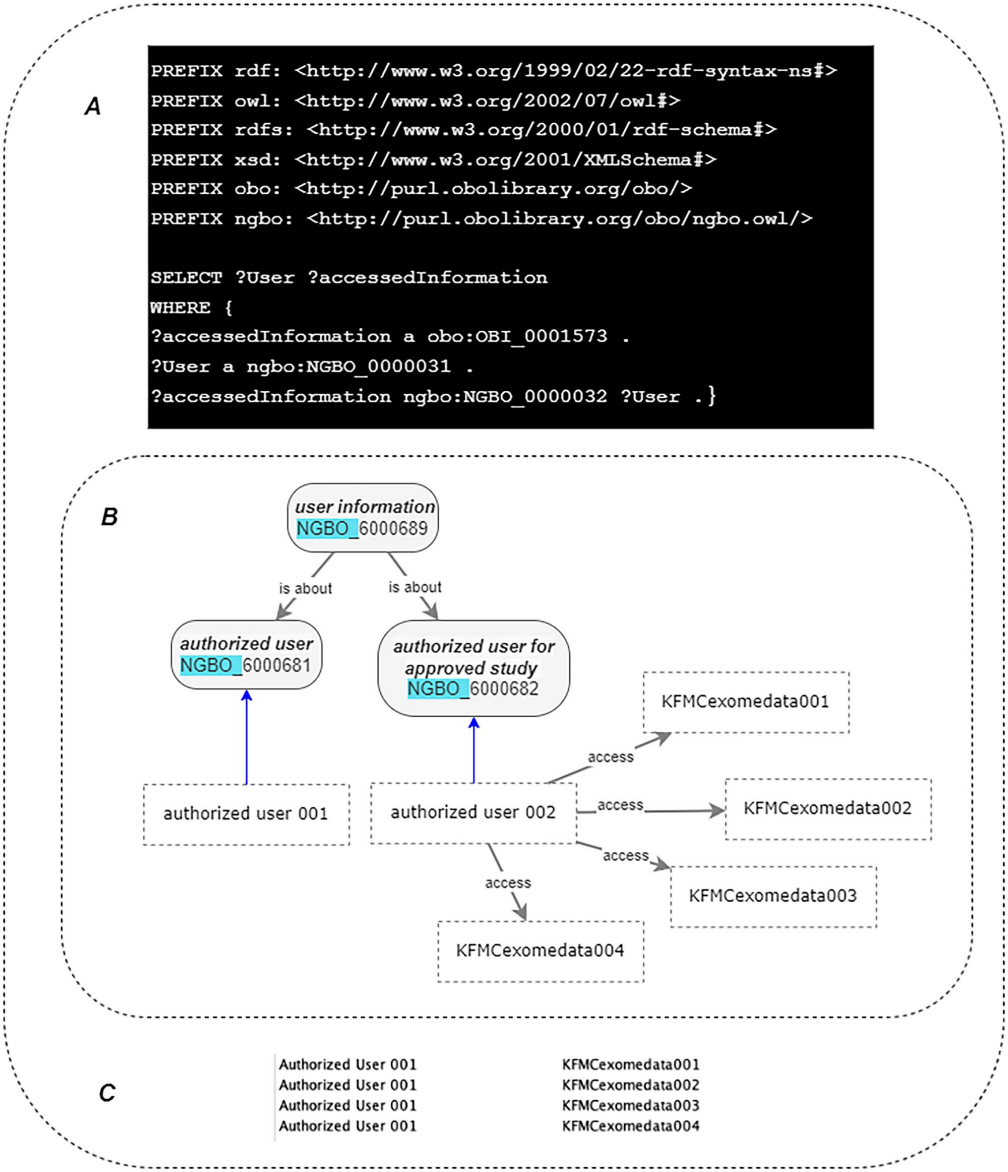
Using NGBO for data privacy and user access controls. (A) A SPARQL query to find who can access a set of exome files. (B) An example of relations between individuals. (C) SPARQL query result, all individuals were linked to the set of exam data files through the relation “access by” selected in the variable? User. Here, “authorized user 002” has access to the approved data sets through “access,” while “Authorized user 001” cannot access the same dataset, as there is no relationship established between the user and the dataset. This example is for demonstration purposes; in a production-level security system, access control would typically involve group-based access, project-level permissions, and control over all content within specific projects.

When dealing with biobanking data, organizations must ensure justified data access for the right person. In this work, we illustrate the concept using a graph-based representation of contextual data with relational links, introducing four classes (“data owner,” “guest,” “authorized user,” and “authorized user for approved study”) to demonstrate how semantic relations can manage access protocols via the “accessed by” object property. This example fulfills the CAP requirements for traceability. However, it is intended as a demonstration. In a production-level security system, access control would typically involve group-based access, project-level permissions, and control over all content within specific projects. Figure 6 shows an example of a SPARQL Protocol and RDF Query Language (SPARQL) query used to find all users accessing exome data files.”

## 4 Discussion

The findings from the SHGP and R2 Health Cluster use cases demonstrate NGBO’s practical value in enhancing data integration, discoverability, and metadata consistency. In SHGP, NGBO replaced manual data searches with ontology-driven querying, resolving inconsistencies in specimen metadata and enabling structured retrieval of sequencing data. In the R2 Health Cluster, NGBO standardized metadata across multiple grant-funded projects, reducing inconsistencies in key fields such as gene symbols and variant formats. Feedback from the data governance team noted a reduction in manual curation time and improved readiness for cross-study comparisons, affirming NGBO’s role in advancing semantic interoperability.

NGBO makes a distinct contribution to the biobanking domain by modeling omics contextual data alongside specimen and process metadata—something not fully addressed by existing ontologies. It unifies wet bench, sequencing, and analysis processes using standardized, interoperable terms, while supporting traceability and CAP-compliant documentation. This allows NGBO to bridge the gap between traditional biobanking systems and the demands of modern, data-intensive omics research.

Aligned with FAIR principles, NGBO improves data findability through standardized terms registered in the OLS, accessibility via consistent identifiers, and interoperability by linking with ontologies such as OBIB and OBI. Its use of logical constraints and structured annotations enhances reusability by reducing ambiguity and ensuring quality.

NGBO also supports long-term archiving and structured retrieval of data and contextual metadata. Using NGBO identifiers, data files, and associated information can be accessed via standardized query protocols, enabling federated queries across biobank systems.

While the current version demonstrates successful integration and querying in SHGP and R2, broader deployment is needed to validate its performance in diverse biobank environments. NGBO does not yet include automated integration with Electronic Health Records (EHR) systems or dynamic privacy layers. Future development will explore adaptive access control mechanisms that apply sensitivity-based privacy settings across datasets, fulfilling real-world data governance needs.

Finally, NGBO lays the groundwork for future applications in clinical research. By enabling integration with external resources such as ClinVar, it can support genomic annotation within clinical workflows. This semantic linkage between biobank and clinical datasets has the potential to significantly enhance personalized medicine and bridge the gap between research and clinical decision-making. Future work may explore automating data annotation using large language models and retrieval-augmented generation (RAG) tools. For example, RefAI (Li *et al.* 2024), a GPT-powered system for biomedical literature retrieval and summarization, demonstrates how RAG methods can improve the precision and scalability of information retrieval. Adapting similar approaches to semantic data mapping in biobanking contexts could further extend NGBO’s utility by enabling intelligent, automated ontology-based annotation and querying at scale.

## Supplementary Material

vbaf131_Supplementary_Data

## Data Availability

NGBO is freely available at https://github.com/Dalalghamdi/NGBO, and accessible from OLS https://www.ebi.ac.uk/ols4/ontologies/ngbo.
