# Supplementary material for: Next generation biobanking ontology: introducing–omics contextual data to biobanking ontology
Source: Bioinform Adv. 2025 Aug 7;5(1):vbaf131. doi: 10.1093/bioadv/vbaf131 (PMC12342351; doi:10.1093/bioadv/vbaf131)

Competency Question 1: Search for disease **x (adenocarcinoma specimen)** omics-derived data **(DNA sequence data)** across all available repositories.

Example Query 1:


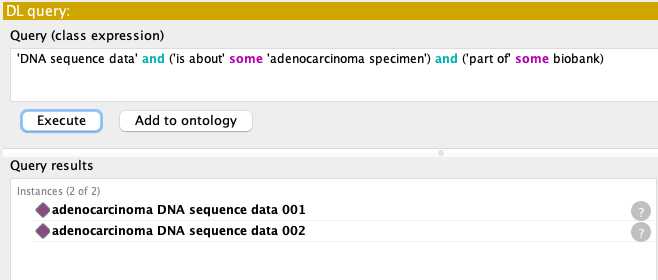


Competency Question 2:

Search for disease **x (adenocarcinoma specimen)** omics-derived data **y (DNA sequence data)**  available in repositories **a, b (King Fahad Medical City biobank & King Faisal Specialist Hospital)**  with a mutation in gene **z (KRAS mutation)**.

Example Query 2:

**
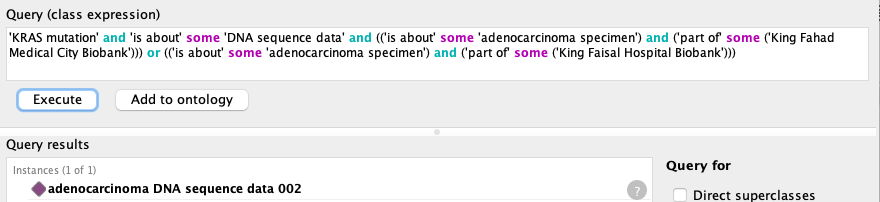
**

Competency Question 3:

Data format **x (fasta files)** for omics-derived data **y (DNA sequencing data)** for disease **z (autism)** filtered by life stage **(pediatric patients)** available in repositories **a (King Fahad Medical City)**

Example Query 3:

**
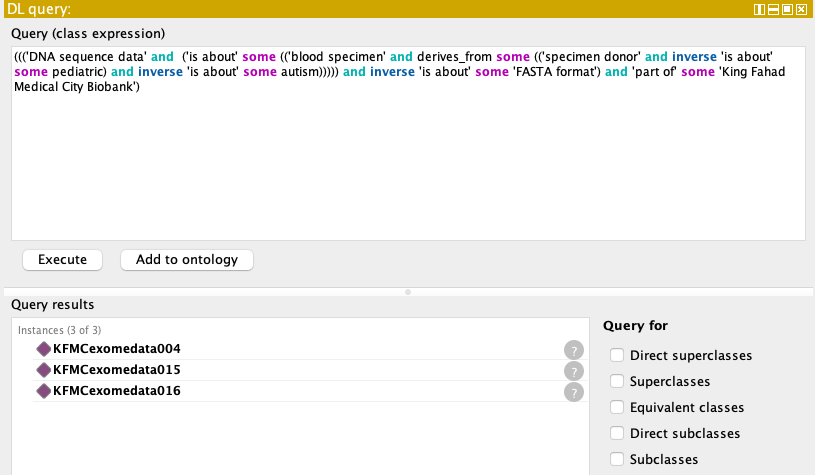
**

Competency Question 4:

Number of data formats **x (fasta files)** for omics-derived data **y (DNA sequencing data)** for disease **z (autism)** filter by life stage **(pediatric patients)** available in repositories **a (King Fahad Medical City)**

Example Query 4:

**
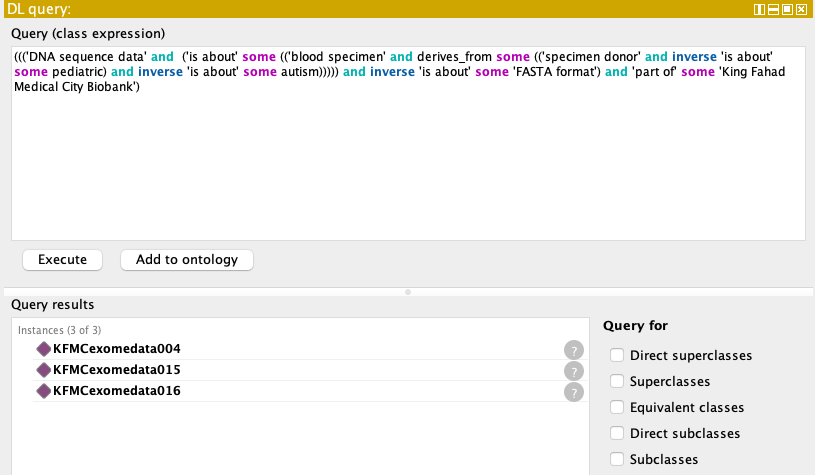
**

Competency Question 5:

Search for omics-derived data for disease **x (bladder cancer)** and subset it by smoking history

**(current reformed smoker for less than 15 years history), (current reformed smoker for more than 15 years history).**

Example Query 5:


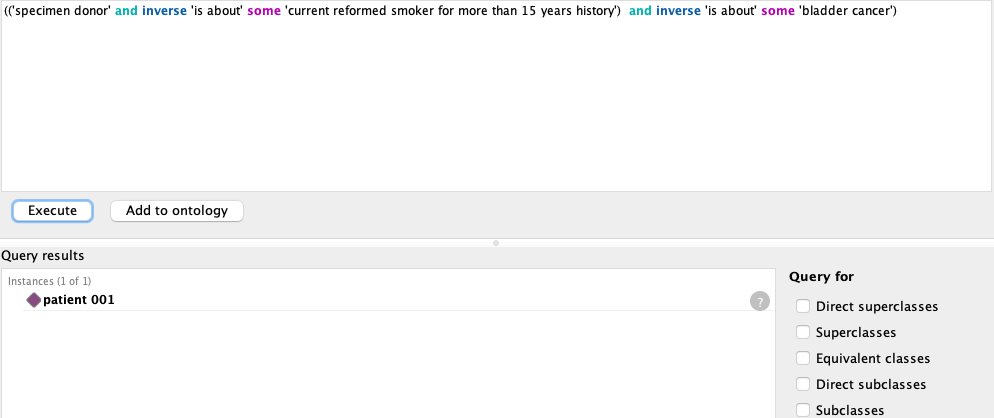


Competency Question 6:

Search for omics-derived data **(DNA sequencing data)** **x** with mutation **y (FGFR3)** and **z (PIK3CA)**

Example Query 6:


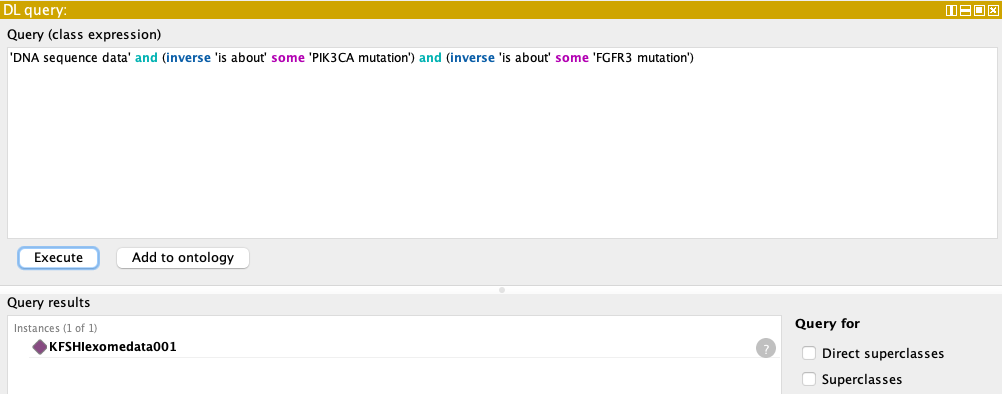

Supplement: vbaf131_Supplementary_Data [file vbaf131_supplementary_data.zip › Supplementary Material CQ.docx]
