# Supplementary figures and images for: Next generation biobanking ontology: introducing–omics contextual data to biobanking ontology
Source: Bioinform Adv. 2025 Aug 7;5(1):vbaf131. doi: 10.1093/bioadv/vbaf131 (PMC12342351; doi:10.1093/bioadv/vbaf131)

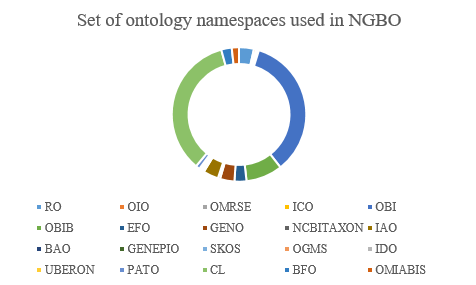

Supplement: vbaf131_Supplementary_Data [file vbaf131_supplementary_data.zip › supplementary figure 1 namesspaces used in NGBO.png]
